# Childbirth Related Post-traumatic Stress Symptoms and Maternal Sleep Difficulties: Associations With Parenting Stress

**DOI:** 10.3389/fpsyg.2018.02103

**Published:** 2018-11-02

**Authors:** Paola Di Blasio, Elena Camisasca, Sarah Miragoli

**Affiliations:** ^1^CRIdee, Università Cattolica del Sacro Cuore, Milan, Italy; ^2^Faculty of Psychology, eCampus University, Novedrate, Italy

**Keywords:** sleep difficulties, postpartum, PTS symptoms, parenting stress, mothers

## Abstract

In the literature, increasing evidence is showing the importance of sleep difficulties in the development or maintenance of posttraumatic stress (PTS) symptoms as well as the association between childbirth-related PTS symptoms and early maternal emotions and perceptions of their children. However, little is known regarding the effects of maternal sleep difficulties on parenting or about the mediational role of childbirth-related PTS symptoms in this association. The present study (pregnancy: T0; 1 month postpartum: T1; 3 months postpartum: T2) had two aims. The first one was to explore whether maternal sleep difficulties could contribute to the maintenance of PTS symptoms and whether PTS symptoms could contribute to the maintenance of maternal sleep difficulties. The second purpose was to explore, at 3 months (T2), the associations among childbirth-related PTS symptoms, maternal sleep difficulties, and the three dimensions of parenting stress [parental distress (PD), parent–child dysfunctional interaction, and difficult child] by examining the mediational role of both maternal sleep difficulties and childbirth-related PTS symptoms. Self-report questionnaires were administered to 95 women at different times (T0, T1, and T2). Mediational results confirmed the bidirectional effects between maternal sleep difficulties and PTS symptoms and their reciprocal role of maintenance of symptoms. Moreover, at 3 months postpartum (T2), sleep difficulties mediated the association between PTS symptoms and the three dimensions of maternal parenting stress, while PTS symptoms mediated the associations among maternal sleep difficulties, PD, and difficult child dimensions of parenting stress. The study contributes to the understanding of the maintenance factors of childbirth-related PTS symptoms and of the relationships among PTS symptoms, maternal sleep difficulties, and parenting stress.

## Introduction

Good sleep quality is a well-recognized predictor of both physical and mental health (Ohayon et al., 2017). Conversely, sleep disorders are frequently linked to concomitant psychiatric illness, particularly anxiety and mood disorders (Britvić et al., 2016). More precisely, sleep disturbances have been defined a hallmark of posttraumatic stress disorder (PTSD), a clinical syndrome typified by re-experiencing, avoidance, and hyperarousal symptoms, which persist for more than 1 month after exposure to a traumatic event (Britvić et al., 2016).

In the literature, there is growing evidence that sleep difficulties following trauma exposure could constitute a specific mechanism involved in the pathophysiology of chronic PTSD, and are not simply an effect or manifestation of the disease (Germain et al., 2008; Williams et al., 2015). Impaired sleep (insufficient or fragmented sleep) may prevent the individual ability to manage stress and recover from traumatic events. More specifically, sleep disturbances may decrease the cognitive resources for managing stress, create a state of hyperarousal, and hinder restorative sleep for recovery from traumatic events (Bryant et al., 2010; Williams et al., 2015).

Empirical evidence has shown that nightmares and insomnia, which are diagnostic symptoms of PTSD, and other sleep disturbances (e.g., nocturnal anxiety attacks, sleep avoidance and terrors, acting out dreams, simple and complex motor behaviors and vocalizations, periodic leg movement disorders, etc.) are frequently observed in PTSD patients (Krakow et al., 2001; Germain et al., 2005). Moreover, in order to better investigate the specific features of sleep disorders associated with PTSD, (Britvić et al., 2016) explored the nature and intensity of sleep disorders in patients with PTSD, compared to in depressed patients. Their findings suggested that PTSD patients went to sleep later, needed two times more time to fall asleep, had more nightmares, got up earlier, and had shorter total durations of sleep than depressive patients.

In the literature, it has become clear that sleep disorders could be a significant predictor of both the development of PTSD and of worse clinical outcomes for the survivors of trauma (for a review, see Lamarche and De Koninck, 2007). More precisely, some studies (Koren et al., 2002; Mellman et al., 2007) have shown that sleep difficulties during the month after a trauma could predict the onset of PTSD symptoms a year after, and that fragmented REM patterns could be associated with PTSD. Other studies have demonstrated that among trauma survivors (combat veterans or civilians who have experienced traumatic events such as natural disaster, violent crime, sexual assault, or critical illness), sleep disorders were correlated with a series of adverse clinical outcomes, including depression, suicidality, and increased alcohol and drug use (Saladin et al., 1995; Krakow et al., 2000, 2002; Nishith et al., 2001).

Despite the growing interest in the association between sleep disturbances and PTSD, to date, only one study has explored the relationship between maternal sleep quality and childbirth-related posttraumatic stress (PTS) symptoms (Garthus-Niegel et al., 2015). More precisely, using a Norwegian cohort of women, Garthus-Niegel et al. (2015) examined whether sleep difficulties, a poor relationship with the baby’s father, and personality could contribute to the maintenance of PTSD symptoms from 8 weeks after birth to 2 years. The results showed that only insomnia symptoms remained significant after controlling for the other factors (i.e., personality, child sleep, social support, and life events) and that these symptoms mediated the effects of PTSD symptoms from 8 weeks to 2 years postpartum.

These results are interesting because they highlighted, for the first time, the significant role of sleep disturbances in the mental health of new parents, who are usually described as chronically sleep deprived (Tikotzky, 2016) due to changes in hormonal and melatonin levels caused by infant sleep/wake patterns and feeding needs.

Many studies have illustrated that sleep during the postpartum period was significantly shorter and disrupted, compared with sleep during pregnancy (Hunter et al., 2009; Montgomery-Downs et al., 2010). Moreover, sleep difficulties lead to increased levels of sleepiness (physiological drive for sleep) and fatigue (weariness and reduced capacity for activity), which may persist into the postpartum period (Tikotzky, 2016).

Regarding the presence of childbirth-related PTS symptoms, the literature (Engelhard et al., 2001; Holditch-Davis et al., 2003) illustrated how not only atypical conditions (preterm birth and/or serious pathological conditions) but even regular childbirth (e.g., childbirth without obstetric/neonatal complication) could affect this clinical condition (Elmir et al., 2010; Andersen et al., 2012; Di Blasio et al., 2015a,b; Simpson and Catling, 2015). It was also suggested that the prevalence of PTSD from regular childbirth ranges from 2.8 to 6.9% (Grekin and O’Hara, 2014; Yildiz et al., 2017). Regarding the stability of PTS symptoms across time, some studies have shown significant stability in the period from 6 weeks to 12 months (White et al., 2006), while others (e.g., Garthus-Niegel et al., 2015) showed that PTSD prevalence went down by approximately 50% (i.e., from 1.9 to 1%).

In the literature, increasing evidence has shown that childbirth-related PTS symptoms may be associated with parenting stress and difficulties regarding early parental emotions and perceptions of their children (Ayers et al., 2006; Camisasca et al., 2017; Cook et al., 2018; for a review, see Davies et al., 2008; Parfitt and Ayers, 2009; Di Blasio et al., 2017). More precisely, avoidant or overanxious emotions due to childbirth-related PTSD were significantly associated to the mothers’ negative perceptions of their children (Ayers et al., 2006; Parfitt and Ayers, 2009). Moreover, Davies et al. (2008) indicated that at 6 weeks postpartum, the mothers with full or sub-threshold PTS symptoms perceived their children as less emotionally “warm” and more demanding and prone to disturb, compared with a non-symptomatic group.

Negative perceptions about the child and his/her behavior are implicit in the construct of parenting stress, which was labeled as the perceived discrepancy between personal resources and contextual demands associated with parenthood (Abidin, 1995). McDonald et al. (2011) carried out a follow-up study on 81 women to explore the prevalence of childbirth-related PTS symptoms at 2 years postpartum and to investigate the relationship between PTS symptoms and both maternal perceptions of the quality of the relationship with their children and their parenting stress. The findings suggested that some women (17.3%) continued to report childbirth-related PTS symptoms 2 years after the childbirth experience. Moreover, women experiencing PTS symptoms at 2 years postpartum reported greater parental distress (PSI-PD subscale), more mother–child dysfunctional interactions (PSI-PCDI subscale), and more perceptions of the child as being more difficult (PSI-DC subscale) than women experiencing lower PTS symptoms. Di Blasio et al. (2017) explored the associations among childbirth-related PTS symptoms (evaluated at 87 h and at 3 months postpartum), parenting stress, and children’s adjustment at 18 months. They also investigated the mediational role of parenting stress dimensions in the relationship between postpartum PTS symptoms and children’s adjustment. The findings illustrated positive associations among PTS symptoms at 3 months, parenting stress, and children’s adjustment. Moreover, at 18 months, parental distress (PD) partially mediated the association between PTS symptoms and children’s internalizing behaviors, while the Difficult Child (DC) dimension of parenting stress fully mediated the effects of PTS symptoms on children’s externalizing behaviors.

In summary, there is growing evidence (Cook et al., 2018) that childbirth-related PTS symptoms could represent a clinically serious condition that is frequently associated with difficulties in mother–infant relationships, specifically with parenting stress. In the literature, although a series of studies have outlined that parenting stress due to children’s disabilities (Davis and Carter, 2008; Gallagher et al., 2010) and chronic illness (Wirrell et al., 2008) could affect parents’ sleep difficulties, to date, the study of the predictive role of parents’ sleep difficulties on parenting stress is a new research direction. Moreover, surprisingly, the role of sleep difficulties, as an important risk factor for dysfunctional parenting, has not been systematically explored (Tikotzky, 2016). An important exception is the study of Tikotzky (2016), who aimed to investigate whether maternal sleep was associated with maternal bonding. The author suggested that maternal sleep difficulties (e.g., higher insomnia scores, more night waking, and longer sleep latency) could be associated with negative maternal perceptions and feelings toward her infant and the mother–infant relationship. The results confirmed the hypothesis, highlighting the importance of understanding the negative implications of maternal sleep problems on parenting.

Built on the above-cited literature, the present study was aimed at advancing knowledge regarding the associations among childbirth-related PTS symptoms, maternal sleep difficulties, and parenting stress. Indeed, although Garthus-Niegel et al. (2015) outlined how insomnia symptoms could constitute a maintaining factor for ongoing PTSD in the postpartum period and Tikotzky (2016) underscored the negative implications of maternal sleep problems on parenting, to date no empirical studies investigated the role of sleep difficulties on maternal parenting stress.

The present research study (pregnancy: T0; 1 month: T1; 3 months: T2) had two aims. The first was to explore the bidirectional effects of maternal sleep difficulties and PTS symptoms. More precisely we were interested to assess whether maternal sleep difficulties (measured at T0 and T1) could contribute to the maintenance of PTS symptoms (measured at T1 and T2) and whether PTS symptoms (measured at T0 and T1) could contribute to the maintenance of maternal sleep difficulties symptoms (measured at T1 and T2). We hypothesized (H1) that sleep difficulties during pregnancy (T0) and 1 month after childbirth (T1) could mediate both the associations between PTS symptoms at T0 and PTS symptoms at T1 and the associations between PTS symptoms at T1 and PTS symptoms at T2. In the same way, we supposed that PTS symptoms during pregnancy (T0) and 1 month after childbirth (T1) could mediate both the associations between maternal sleep difficulties at T0 and maternal sleep difficulties at T1 and the associations between maternal sleep symptoms at T1 and the same symptoms at T2.

In line with the indications of the literature (Krakow et al., 2001; Germain et al., 2005; Lamarche and De Koninck, 2007; Garthus-Niegel et al., 2015), we supposed that both maternal sleep difficulties and PTS symptoms could be significant maintenance factors for ongoing PTS symptoms and sleep difficulties.

Once we explored the bidirectional effects between PTS symptoms and maternal sleep difficulties, our second purpose was to analyze the associations among childbirth-related PTS symptoms, maternal sleep difficulties, and the three dimensions of parenting stress (PD, PCDI, and DC), at 3 months (T2). Indeed, the evaluation, in a single model of analysis, of PTS symptoms and of the maternal sleep difficulties, as risk factor for parenting stress, has not been explored. We assumed that both PTS symptoms and maternal sleep difficulties could be strictly associated with parenting stress at 3 months postpartum (H2). Moreover, on the basis of the literature that outlined how sleep difficulties could both contribute to the maintenance of PTSD symptoms (Garthus-Niegel et al., 2015) and be an effect or manifestation of this disease (Krakow et al., 2001; Germain et al., 2005), we hypothesized (H3) that maternal sleep disturbances (T2) could mediate the association between childbirth-related PTS symptoms (T1) and maternal parenting stress (T2), and that maternal childbirth-related PTS symptoms (T2) could also mediate the association among maternal sleep difficulties, symptoms (T1), and maternal parenting stress (T2).

In other words, we were interested in exploring the potential bidirectional effects of maternal sleep difficulties and childbirth-related symptoms, in their associations with maternal parenting stress.

## Materials and Methods

### Participants

Ninety-five women between the 32nd and 40th weeks of pregnancy (T1) were recruited during prenatal courses and at hospitals in one Northern Italian city (Milan). The research protocol, approved by the Institutional Review Board, was presented individually to each participant. Spontaneous and unpaid consent to participate was obtained. The informed consent form described the project, its goals, the measures, and the voluntary nature of participation. The participants were 95 women, aged 20–41 years (*M* = 32.62, *SD* = 3.79). Most of them were Italian (97%), employed (89%), and married or cohabitating (98%), and had a medium-high level of education: 57.3% with a high-school certificate (13 years of education), 37.9% with a degree (17 years), 2.9% with a middle-school certificate (8 years), and 1.9% with a Ph.D. (19 years). The newborns were 52% female and 48% male, and most of the women were primiparous (85.4%). Compared with the ISTAT National Demographic Statistics (2017), the women in the study were the same age (mean age of 32.6 years vs. 32.4 years) and were less often single (2% vs. 5.1%).

Regarding the obstetric characteristics, 75% of the women had a full-term pregnancy with spontaneous natural childbirth; in particular, 25% did not receive epidural analgesia, and 50% did receive epidural analgesia. Moreover, 8% of the women had a planned cesarean section, and 17% had an emergency cesarean section. At birth, the Apgar score (at 1 min after delivery) was normal in 92% of the cases and moderately depressed in 2% of cases, while the data were missing for 6% of the cases.

### Procedure

The first phase of this study took place during antenatal courses or medical checks between the 32nd and 40th weeks of pregnancy (T0). One hundred and forty-two women were approached, of whom spontaneous and unpaid consent to participate was obtained from 120 women (84.5%). After each participant provided informed consent, she completed the questionnaires assessing her demographic characteristics, sleep disturbances (Pittsburgh Sleep Quality Index; Buysse et al., 1989), and PTS symptoms (Los Angeles Symptoms Checklist; King et al., 1995). The second and third phases took place at 1 month (T1) and at 3 months (T2), and 105 women (refusal rate: 12.5%) and 95 women (refusal rate: 9.5%), respectively, were reassessed with the abovementioned measures plus the Parenting Stress Index-Short Form (Abidin, 1995; Guarino et al., 2008), which was added at T2. This study was carried out in accordance with the recommendations of the Declaration of Helsinki and with the Ethical AIP Guidelines. The protocol was approved by the Ethics Committee of the Catholic University of Milan (12.06.2014). All subjects gave written informed consent in accordance with the Declaration of Helsinki.

### Measures

#### Pittsburgh Sleep Quality Index (PSQI)

The PSQI (Buysse et al., 1989) is utilized as a global index of sleep quality during the past month and is a self-report measure of 19 items (scored from 0 to 3). An overall sleep quality score is computed (range 0–21) by summing seven subscale scores (range 0–3): *subjective sleep quality*, *sleep latency*, *sleep duration*, *habitual sleep efficiency*, *sleep disturbances*, *use of sleeping medication*, and *daytime dysfunction*. The sum of the subscale scores generates one global score: Higher PSQI values indicate worse sleep quality. For the Italian validation (Curcio et al., 2013), the PSQI showed an overall reliability coefficient (Cronbach’s α) of 0.835, indicating a high degree of internal consistency. In the current sample, the PSQI showed acceptable reliability (α = 0.73).

#### Los Angeles Symptom Checklist (LASC)

The LASC (King et al., 1995) is a self-report measure of 43 items designed to measure a broad range of anxiety symptoms. Of the 43 items, 17 capture re-experiencing (nightmares; vivid memories of unpleasant prior experiences), avoidance and numbing (avoidance of activities that remind one of prior unpleasant experiences; feeling emotionally numb), and hyperarousal symptoms (irritability, difficulty falling asleep) of PTSD. Each item is a word or phrase that is rated on a 5-point Likert scale ranging from 0 (*no problem*) to 4 (*extreme problem*), reflecting the extent to which the particular symptom was a problem for the respondent during the past month. The sum of all 43 items (ranging from 0 to 172) assesses the difficulty in adaptation (distress index) as an effect of traumatic experiences, while the sum of the 17 aforementioned items assesses the gravity of PTSD (severity index). In this study, during pregnancy (T0), the participants were asked to specifically think about their worst past negative experience, when filling out the LASC questionnaire. Both at 1 and 3 months postpartum (T1 and T2), women were asked to specifically think about the delivery experience when filling out the LASC questionnaire. In this study, only the PTSD severity index was used. The cut-off score index for PTSD severity index was 25.26, as suggested by King et al. (1995, p. 14).

#### Parenting Stress Index-Short Form (PSI-SF)

The PSI-SF (Abidin, 1995; Italian validation by Guarino et al., 2008) is a self-report measure for parents of children aged 1 year. The 36 items of the questionnaire are rated on a 5-point scale, ranging from *strongly agree* to *strongly disagree*. It is composed of four subscales: PD (12 items), DC (12 items), P-CDI (12 items), and a defensive responding subscale that consists of seven items drawn from the PD subscale. The Defensive Responding subscale measures parental bias in reporting by quantifying the wish of parents to present a favorable conception of themselves and minimize difficulties in their parent–child relationship. The PD (e.g., “I often feel I cannot handle things well”) subscale measures the parents’ sense of competence/incompetence in rearing the child, discord with the partner, lack of social support, and stress associated with the restrictions deriving from their parental role. The Parent–Child Dysfunctional Interaction (P-CDI; e.g., “My child rarely does things for me that make me feel good”) subscale focuses on the parents’ perceptions in terms of the emotional quality of their relationship with their children. The Difficult Child (DC; e.g., “My child seems to cry or fuss more often than most children”) subscale measures the parent’s perception of their children in terms of temperament, requesting and provoking behaviors, and non-collaborative and demanding behaviors. This subscale shows how easy or difficult the parent perceives his/her child as being. According to Abidin (1995), the scores on the DC scale could have different interpretations based on the child’s age. For a child who is less than 18 months old, parent scores at or above the 90th percentile would indicate that the child could be having problems with self-regulatory processes. These could be physical or temperamental problems. For a child who is 2 years old or older, the parent may be having a hard time gaining his or her child’s cooperation and/or managing the child’s behavior.

The sum of the three subscales (PD, P-CDI, and DC) allows for obtaining the total stress score, which indicates the overall level of the stress associated with the parental role not deriving from other roles or events. A total PSISF score at the 90th percentile represents a clinically significant level of parenting stress (Abidin, 1995) and can be used as an indicator that counseling or other support is required. The internal consistency values of the Italian validation of the PSI-SF (Guarino et al., 2008) were: α = 0.91 for the Total Stress scale, α = 0.91 for the PD subscale, α = 0.95 for the P-CDI subscale, and α = 0.90 for the DC subscale. For the purposes of this study, we used the PD, P-CDI, and DC subscales, with internal consistency values of α = 0.89 for the Total Stress scale, α = 0.82 for the PD subscale, α = 0.81 for the P-CDI subscale, and α = 0.83 for the DC subscale.

### Analysis Strategy

**FIGURE 1:**
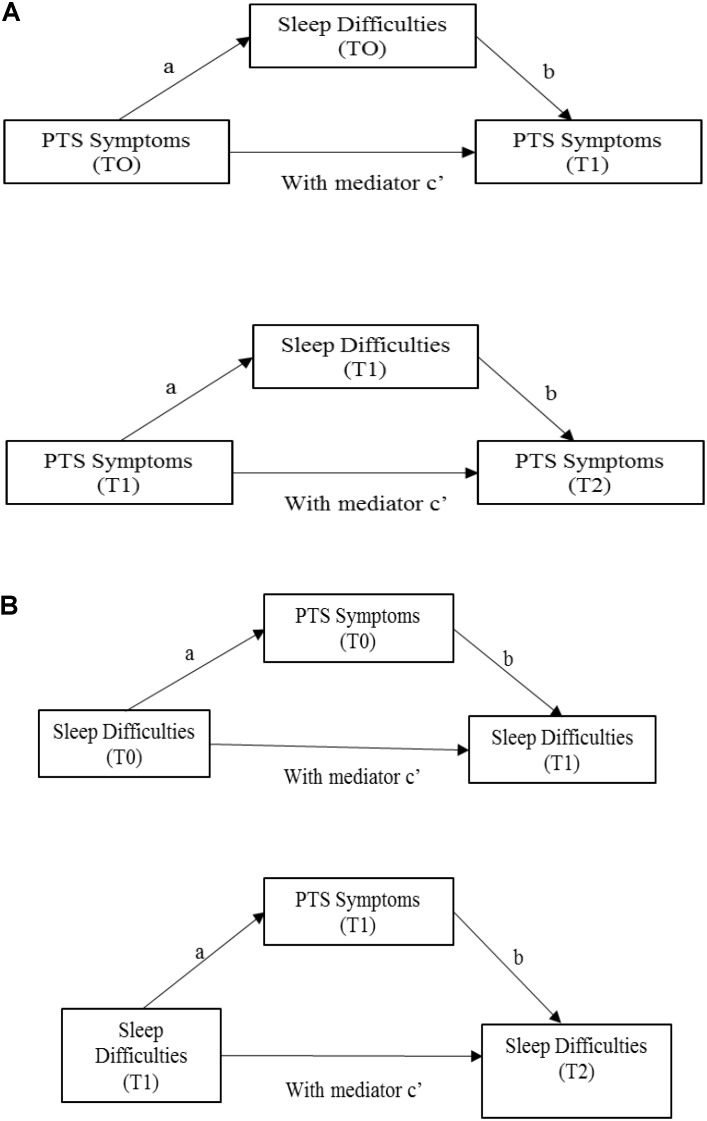
First aim: mediational analyses. **(A)** First and second mediation models. **(B)** Third and fourth mediation models. Path a represents the effect of X on the proposed mediator, whereas path b is the effect of M on Y partialling out the effect of X. The indirect effect of X on Y through M can then be quantified as the product of a and b (i.e., ab). The total effect of X on Y can be expressed as the sum of the direct and indirect effects: c = c′+ab. Equivalently, c′ is the difference between the total effect of X on Y and the indirect effect of X on Y through M, that is, c′ = c-ab (Preacher and Hayes, 2008, p. 880).

Descriptive statistics were computed for all of the variables. Pearson’s *r* correlations were used to investigate the associations between the variables. Regarding the first aim of the study, two mediation models were utilized, using the Process Macro for SPSS (Hayes, 2012), applying Model 4 with 10,000 bias-corrected bootstrap samples (Figure 1). The bias-corrected bootstrapping resampling method is particularly suitable for small samples (Hayes, 2012). Our preference is based on the fact that other methods for testing indirect effects assume a standard normal distribution when calculating the *p*-value for the indirect effect, whereas bootstrapping does not assume normality of the sampling distribution. In addition, the bootstrap method repeatedly samples from the dataset, estimating the indirect effects with each resampled dataset. This process is repeated 10,000 times, producing bias-corrected accelerated confidence intervals for the indirect effect (Hayes, 2012).

**FIGURE 2:**
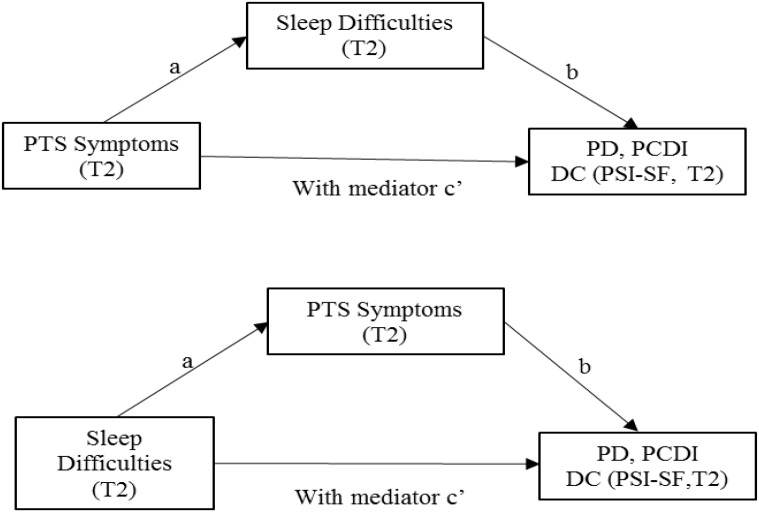
Second aim: mediational analyses. Path a represents the effect of X on the proposed mediator, whereas path b is the effect of M on Y partialling out the effect of X. The indirect effect of X on Y through M can then be quantified as the product of a and b (i.e., ab). The total effect of X on Y can be expressed as the sum of the direct and indirect effects: c = c′+ab. Equivalently, c′ is the difference between the total effect of X on Y and the indirect effect of X on Y through M, that is, c′ = c-ab (Preacher and Hayes, 2008, p. 880).

In the first mediation model, maternal PTS symptoms at T0 were added as the predictor, along with maternal PTS symptoms at T1 as an outcome and maternal sleep difficulties at T0 as a mediator. In the second mediation model, maternal PTS symptoms at T1 were added as the predictor, along with maternal PTS symptoms at T2 as an outcome and maternal sleep difficulties at T1 as a mediator (Figure 1A). Moreover, in the third mediation model, maternal sleep difficulties at T0 were added as the predictor, along with maternal sleep difficulties at T1 as an outcome and maternal PTS symptoms at T0 as a mediator. In the fourth mediation model, maternal sleep difficulties sat T1 were added as the predictor, along with maternal sleep symptoms at T2 as an outcome and maternal PTS symptoms at T1 as a mediator (Figures 1B, 2).

Regarding the second aim of the study, two series of mediation models were utilized, using the Process Macro for SPSS (Hayes, 2012). In the first set of mediation models, PTS symptoms at T2 were added as the predictor, along with maternal dimensions of parenting stress (PD, PCDI, and DC) as outcomes and maternal sleep difficulties at T2 as a mediator. In the second set of mediation models, maternal sleep difficulties at T2 were added as the predictor, along with maternal dimensions of parenting stress (PD, PCDI, and DC) as outcomes and maternal PTS symptoms at T2 as a mediator.

## Results

### Descriptive and Correlational Analyses

The descriptive and correlational analyses are presented in Table 1. Regarding PTS symptoms, the data also showed that 2.3% of mothers had PTSD during pregnancy (T0), while 3.1% of the mothers had PTSD at 1 month (T1) and 3 months postpartum.

**Table 1:** Correlational analyses.

	1	2	3	4	5	6	7	8	9	10	*M* (*SD*)
(1) PTS total symptoms (T0)	1										4.30 (7.31)
(2) PTS total symptoms (T1)	0.688^∗∗^	1									6.03 (7.20)
(3) PTS total symptoms (T2)	0.725^∗∗^	0.603^∗∗^	1								6.27 (6.20)
(4) Sleep difficulties (T0)	0.543^∗∗^	0.514^∗∗^	0.490^∗∗^	1							5.85 (3.0)
(5) Sleep difficulties (T1)	0.362^∗∗^	0.455^∗∗^	.480^∗∗^	0.601^∗∗^	1						7.25 (3.2)
(6) Sleep difficulties (T2)	0.363^∗∗^	0.280^∗∗^	0.389^∗∗^	0.219^∗^	0.322^∗∗^	1					5.98 (3.2)
(7) PSI-parental distress (T2)	0.538^∗∗^	0.478^∗∗^	0.596^∗∗^	0.254^∗^	0.375^∗∗^	0.514^∗∗^	1				23.27 (7.9)
(8) PSI-parent child dysfunctional interaction (T2)	0.171	0.104	0.235^∗^	-0.001	0.043	0.448^∗∗^	0.487^∗∗^	1			16.7 (4.7)
(9) PSI-difficult child (T2)	0.348^∗∗^	0.278^∗∗^	0.392^∗∗^	0.097	0.184	0.580^∗∗^	0.683^∗∗^	0.588^∗∗^	1		19.27 (6.7)
(10) PSI-total stress (T2)	0.450^∗∗^	0.375^∗∗^	0.513^∗∗^	0.163	0.273^∗∗^	0.607^∗∗^	0.898^∗∗^	0.732^∗∗^	0.901^∗∗^	1	58.69 (16.91)


*^∗^*p* < 0.05, ^∗∗^*p* < 0.01.*

More specifically, during T0, the mean scores of the three LASC subscales (Intrusive Symptoms, I; Avoidance Symptoms, A; and Hyperarousal Symptoms, H) and of the Severity Index were: I symptoms = 1.14; *A* = 1.55; *H* = 2.23; Severity Index = 4.30. During *T1* the means scores were: *I* = 1.54; *A* = 2.35; *H* = 3.13; Severity Index = 6.03. During T2, the mean scores were: *I* = 1.31; *A* = 2.16; *H* = 3.05; Severity Index = 6.27.

Correlational results illustrated how maternal PTS symptoms at T0 were significantly associated (*r* from 0.69 to 0.73) with PTS symptoms (at T1 and T2); sleep difficulties, both during pregnancy (T0) and at 1 month postpartum (T1), were significantly associated with PTS symptoms at 1 month and at 3 months (*r* from 0.49 to 0.54). Moreover, at 3 months postpartum (T2), sleep difficulties were correlated with PTS symptoms (*r* = 0.39) and with maternal parenting stress (*r* from 0.45 to 0.61); and PTS symptoms were significantly associated with maternal parenting stress (*r* from 0.24 to 0.60).

### Maternal Sleep Difficulties as a Maintaining Factor for Ongoing PTS Symptoms

Two mediational analyses were performed to investigate the first hypothesis of the study (H1): that sleep difficulties during pregnancy (T0) and 1 month after childbirth (T1) could mediate both the association between PTS symptoms at T0 and PTS symptoms at T1 and the association between PTS symptoms at T1 and PTS symptoms at T2.

For the first mediation model, maternal PTS symptoms at T0 were added as a predictor, along with maternal PTS symptoms at T1 as an outcome and maternal sleep difficulties T0 as a mediator. In the second mediation model, maternal PTS symptoms at T1 were added as a predictor, along with maternal PTS symptoms at T2 as an outcome and maternal sleep difficulties at T1 as a mediator. The results (see Table 2) showed that the total effect of PTS symptoms at T0 on PTS symptoms at T1 was significant (Coeff. = 0.68, *p* < 0.001; *c path*). The effect of PTS symptoms at T0 on maternal sleep difficulties at T0 was also significant (Coeff. = 0.23; *p* < 0.001; *a path*). The effect of the mediator on PTS symptoms at T1 was significant (Coeff. = 0.46; *p* < 0.05; *b path*). The direct effect of PTS symptoms at T0 on the outcome (PTS symptoms at T1) was also significant (Coeff. = 0.58; *p* < 0.001; *c*′ *path*). The indirect effect of PTS symptoms at T0 on those at T1, through the mediation of sleep difficulties, was significant (*r*^2^ = 0.47; *F* = 83.51; *p* < 0.001; Coeff. = 0.10; 95% CI = [0.02, 0.23]) and the coefficient of the *R*-squared mediation effect size was 0.23 (95% CI = 0.12, 0.38).

**Table 2:** Sleep difficulties as mediators.

	Coefficient	*SE*	Bootstrap 95% CI	Model *R*^2^	(*p*)
*DV: PTS symptoms at 1 month*				0.47	<0.001
PTS symptoms (at T0)					
Total effect	0.68	0.07			<0.001
Direct effect	0.58	0.08			<0.05
Indirect effect via mediator					
Mothers’ sleep difficulties (at T0)	0.10	0.05	0.02; 0.23		

*DV: PTS symptoms at 3 months*				0.36	<0.001

PTS symptoms (at T1)					
Total effect	0.53	0.07			<0.001
Direct effect	0.43	0.07			<0.001
Indirect effect via mediator					
Mothers’ sleep difficulties (at T1)	0.10	0.04	0.02; 0.22		


For the second model (see Table 2), the results showed that the total effect of PTS symptoms at T1 on PTS symptoms at T2 was significant (Coeff. = 0.53 *p* < 0.001; *c path*). The effect of PTS symptoms at T1 on maternal sleep difficulties at T1 was also significant (Coeff. = 0.22; *p* < 0.001; *a path*). The effect of the mediator on PTS symptoms at T2 was significant (Coeff. = 0.46; *p* < 0.01; *b path*). The direct effect of PTS symptoms at T1 on the outcome (PTS symptoms at T2) was also significant (Coeff. = 0.43; *p* < 0.001; *c*′ *path*). The indirect effect of PTS symptoms at T1 on those at T2, through the mediation of sleep difficulties, was significant (*r*^2^ = 0.36; *F* = 53.00; *p* < 0.001; Coeff. = 0.10; 95% CI = [0.02, 0.22]) and the coefficient of the *R*-squared mediation effect size was 0.18 (95% CI = 0.07, 9.30). Therefore, the results showed that maternal sleep difficulties mediated the associations between PTS symptoms measured at different times and were a significant maintenance factor for ongoing PTS symptoms.

### PTS Symptoms as a Maintaining Factor for Ongoing Maternal Sleep Difficulties

Other two mediational analyses were performed to investigate whether PTS symptoms during pregnancy (T0) and 1 month after childbirth (T1) could mediate both the associations between maternal sleep difficulties at T0 and maternal sleep difficulties at T1 and the associations between maternal sleep symptoms at T1 and the same symptoms at T2 (H1).

For the third mediation model, maternal sleep difficulties at T0 were added as a predictor, along with maternal sleep symptoms at T1 as an outcome and maternal PTS symptoms at T0 as a mediator. In the second mediation model, maternal sleep difficulties symptoms at T1 were added as a predictor, along with maternal sleep symptoms at T2 as an outcome and maternal PTS symptoms at T1 as a mediator. The results (see Table 3) showed that the total effect of maternal sleep difficulties at T0 on maternal sleep difficulties at T1 was significant (Coeff. = 0.70, *p* < 0.001; *c path*). The effect of maternal sleep difficulties at T0 on maternal PTS symptoms at T0 was also significant (Coeff. = 1.2; *p* < 0.001; *a path*). The effect of the mediator on maternal sleep difficulties at T1 was significant (Coeff. = 0.26; *p* < 0.05; *b path*). The direct effect of maternal sleep difficulties at T0 on the outcome (sleep symptoms at T1) was also significant (Coeff. = 0.66; *p* < 0.001; *c*′ *path*). The indirect effect of maternal sleep difficulties at T0 on those at T1, through the mediation of PTS symptoms, was significant (*R*^2^ = 0.36; *F* = 52.55; *p* < 0.001; Coeff. = 0.08; 95% CI = [0.02, 0.23]) and the coefficient of the *R*-squared mediation effect size was 0.12 (95% CI = 0.01, 0.27).

**Table 3:** PTS symptoms as mediators.

	Coefficient	*SE*	Bootstrap 95% CI	Model *R*^2^	(*p*)
*DV: Sleep difficulties at 1 month*				0.36	<0.001
Sleep difficulties (at T0)					
Total effect	0.70	0.09			<0.001
Direct effect	0.66	0.11			<0.001
Indirect effect via mediator					
PTS symptoms (at T0)	0.08	0.06	0.02; 0.23		

*DV: Sleep difficulties at 3 months*				0.10	<0.05

Sleep difficulties (at T1)					
Total effect	0.30	0.09			<0.001
Direct effect	0.23	0.10			<0.05
Indirect effect via mediator					
PTS symptoms (at T1)	0.07	0.03	0.01; 0.17		


For the fourth model (see Table 3), the results showed that the total effect of maternal sleep difficulties at T1 on maternal sleep symptoms at T2 was significant (Coeff. = 0.30; *p* < 0.001; *c path*). The effect of maternal sleep symptoms at T1 on PTS symptoms at T1 was also significant (Coeff. = 0.92; *p* < 0.001; *a path*). The effect of the mediator on maternal sleep symptoms at T2 was significant (Coeff. = 0.23; *p* < 0.05; *b path*). The direct effect of maternal sleep symptoms at T1 on the outcome (maternal sleep symptoms at T2) was also significant (Coeff. = 0.23; *p* < 0.01; *c*′ *path*). The indirect effect of maternal sleep symptoms at T1 on those at T2, through the mediation of PTS symptoms, was significant (*R*^2^ = 0.10; *F* = 10.78; *p* < 0.001; Coeff. = 0.07; 95% CI = [0.01, 0.17]) and the coefficient of the *R*-squared mediation effect size was 0.06 (95% CI = 0.01, 0.15).

Therefore, the results showed that maternal PTS symptoms mediated the associations between maternal sleep difficulties measured at different times and were a significant maintenance factor for ongoing maternal sleep difficulties.

### Maternal Sleep Difficulties as Mediators of the Associations Between Childbirth PTS Symptoms and Parenting Stress

A mediational analysis using Preacher and Hayes’s (2008) approach was performed to verify the indirect effects of PTS symptoms on maternal parenting stress (PD, parent–child dysfunctional interaction, and difficult child) at 3 months postpartum (T2), through the mediation of maternal sleep difficulties. The PTS symptoms were included in the model as an independent variable, in addition to sleep difficulties as a mediator and each PSI-SF subscale as dependent variables.

Regarding the Parental Distress subscale, the results (see Table 4) indicated that the total effect (*c path*) of PTS symptoms on this dimension of parenting stress (Coeff. = 0.72; *p* < 0.001) was significant. The results also illustrated that sleep disturbances were significantly associated with the mediator sleep difficulties (Coeff. = 0.20; *p* < 0.001; *a path*). The mediator’s effect on PD was significant (Coeff. = 0.77; *p* < 0.001; *b path*). The direct effect of sleep disturbances on PD was also significant (Coeff. = 0.56; *p* < 0.001; *c*′ *path*). Moreover, maternal sleep difficulties mediated the association between PTS symptoms and PD (*r*^2^ = 0.35; *F* = 51.30; *p* < 0.001; Coeff. = 0.15; 95% CI = [0.06, 0.30]) and the coefficient of the *R*-squared mediation effect size was 0.17 (95% CI = 0.06, 0.30).

**Table 4:** Sleep difficulties as mediators.

	Coefficient	*SE*	Bootstrap 95% CI	Model *R*^2^	(*p*)
*DV: Parental distress*				0.35	<0.001
PTS symptoms (at T2)					
Total effect	0.72	0.10			<0.001
Direct effect	0.56	0.10			<0.01
Indirect effect via mediator:					
Sleep difficulties (at T2)	0.15	0.05	0.06; 0.30		

*DV: Parent–child dysfunctional interaction*				0.05	<0.05

PTS symptoms (at T2)					
Total effect	0.14	0.06			<0.05
Direct effect	0.04	0.06			>0.05
Indirect effect via mediator:					
Sleep difficulties (at T2)	0.09	0.03	0.04; 0.19		

*DV: Difficult child*				0.15	<0.001

PTS symptoms (at T2)					
Total effect	0.40	0.09			<0.001
Direct effect	0.20	0.09			<0.05
Indirect effect via mediator:					
Sleep difficulties (at T2)	0.20	0.09	0.19; 0.35		


Regarding parent–child dysfunctional interaction (see Table 4), the total effect (*c path*) of sleep disturbances on this dimension of parenting stress (Coeff. = 0.14; *p* < 0.05) was significant. The results also indicated that PTS symptoms had predictive effects on the mediator maternal sleep difficulties (Coeff. = 0.20; *p* < 0.001; *a path*). The mediator’s effect on this dimension of parenting stress was significant (Coeff. = 0.49; *p* < 0.001; *b path*), but the direct effect of PTS symptoms on parent–child dysfunctional interaction was not significant (Coeff. = 0.04; *p* > 0.05; *c*′ *path*). Moreover, sleep difficulties mediated the association between PTS symptoms and parent–child dysfunctional interaction (*r*^2^ = 0.05; *F* = 5.42; *p* < 0.05; Coeff. = 0.09; 95% CI = [0.04, 0.19]) and the coefficient of the *R*-squared mediation effect size was 0.10 (95% CI = 0.00, 0.13).

Regarding the Difficult Child subscale (see Table 4), the results indicated that the total effect (*c path*) of PTS symptoms on this dimension of parenting stress (Coeff. = 0.40; *p* < 0.001) was significant. The results also indicated that PTS symptoms had predictive effects on the mediator sleep difficulties (Coeff. = 0.20; *p* < 0.001; *a path*). The mediator’s effect on the difficult child dimension was significant (Coeff. = 0.99; *p* < 0.001; *b path*). The direct effect of sleep disturbances on the difficult child dimension was also significant (Coeff. = 0.20; *p* < 0.05; *c*′ *path*). Moreover, PTS symptoms partially mediated the association between sleep disturbances and the difficult child dimension (*r*^2^ = 0.15; *F* = 16.89; *p* < 0.001; Coeff. = 0.20; 95% CI = [0.19, 0.35]) and the coefficient of the *R*-squared mediation effect size was 0.12 (95% CI = 0.02, 0.24).

### PTS Symptoms as Mediators of the Associations Between Sleep Difficulties and Parenting Stress

A mediational analysis using Preacher and Hayes’s (2008) approach was performed to verify the indirect effects of maternal sleep difficulties on maternal parenting stress (PD, parent–child dysfunctional interaction, and difficult child) at 3 months postpartum (T2), through the mediation of PTS symptoms. Sleep difficulties were included in the model as an independent variable, along with the PTS symptoms as a mediator and each PSI-SF subscale as dependent variables.

Regarding the Parental Distress subscale, the results (see Table 5) indicated that the total effect (*c path*) of sleep disturbances on this dimension of parenting stress (Coeff. = 1.20; *p* < 0.001) was significant. The results also illustrated that sleep disturbances were significantly associated with the mediator PTS symptoms (Coeff. = 0.85; *p* < 0.01; *a path*). The effect of the mediator on PD was significant (Coeff. = 1.20; *p* < 0.001; *b path*). The direct effect of sleep disturbances on PD was also significant (Coeff. = 0.66; *p* < 0.01; *c*′ *path*). Moreover, PTS symptoms mediated the association between sleep disturbances and PD (*r*^2^ = 0.48; *F* = 42.80; *p* < 0.001; Coeff. = 0.54; 95% CI = [0.22, 0.83]) and the coefficient of the *R*-squared mediation effect size was 0.17 (95% CI = 0.06, 0.30).

**Table 5:** Maternal PTS symptoms as mediators.

	Coefficient	*SE*	Bootstrap 95% CI	Model *R*^2^	(*p*)
*DV: Parental distress*				0.48	<0.001
Sleep difficulties (at T2)					
Total effect	1.20	0.20			<0.001
Direct effect	0.66	0.19			<0.01
Indirect effect via mediator:					
PTS symptoms (at T2)	0.54	0.14	0.22; 0.83		

*DV: Parent–child dysfunctional interaction*				0.24	<0.001

Sleep difficulties (at T2)					
Total effect	0.50	0.10			<0.001
Direct effect	0.43	0.12			<0.01
Indirect effect via mediator:					
PTS symptoms (at T2)	0.06	0.05	-0.02; 0.16		

*DV: Difficult child*				0.36	<0.001

Sleep difficulties (at T2)					
Total effect	1.14	0.16			<0.001
Direct effect	0.93	0.18			<0.01
Indirect effect via mediator:					
PTS symptoms (at T2)	0.20	0.09	0.01; 0.49		


Regarding parent–child dysfunctional interaction (see Table 5), the total effect (*c path*) of sleep disturbances on this dimension of parenting stress (Coeff. = 0.50; *p* < 0.001) was significant. The results also indicated that sleep disturbances had predictive effects on the mediator PTS symptoms (Coeff. = 0.85; *p* < 0.01; *a path*). The effect of the mediator on this dimension of parenting stress was not significant (Coeff. = 0.07; *p* > 0.05; *b path*). The direct effect of sleep disturbances on parent–child dysfunctional interaction was also significant (Coeff. = 0.43; *p* < 0.01; *c*′ *path*). Moreover, PTS symptoms did not mediate the association between sleep disturbances and parent–child dysfunctional interaction.

Regarding the Difficult Child subscale (see Table 5), the results indicated that the total effect (*c path*) of sleep disturbances on this dimension of parenting stress (Coeff. = 1.14; *p* < 0.001) was significant. The results also indicated that sleep disturbances had predictive effects on the mediator of PTS symptoms (Coeff. = 0.85; *p* < 0.01; *a path*). The mediator’s effect on the difficult child dimension was significant (Coeff. = 0.23; *p* < 0.05; *b path*). The direct effect of sleep disturbances on the difficult child dimension was also significant (Coeff. = 0.93; *p* < 0.01; *c*′ *path*). Moreover, PTS symptoms partially mediated the association between sleep disturbances and the difficult child dimension (*r*^2^ = 0.36; *F* = 27.00; *p* < 0.001; Coeff. = 0.20; 95% CI = 0.01; 0.49) and the coefficient of the *R*-squared mediation effect size was 0.12 (95% CI = 0.02, 0.25).

## Discussion

We investigated maternal sleep difficulties from pregnancy to 3 months postpartum to understand their associations with maternal PTS symptoms and parenting stress. It must be outlined that the study’s focus was on sleep difficulties and PTS symptoms, and not on diagnosed sleep disturbances or PTS disorder. Built upon the indications from the literature, which outlined the effects of maternal sleep difficulties on both ongoing childbirth-related PTS symptoms (Garthus-Niegel et al., 2015) and maternal bonding (Tikotzky, 2016), we carried out the investigation with two aims.

The first one was to explore the bidirectional effects of maternal sleep difficulties and PTS symptoms. More precisely, we explored whether maternal sleep difficulties (measured at T0 and T1) could contribute to the maintenance of PTS symptoms (measured at T1 and T2) and whether PTS symptoms (measured at T0 and T1) could contribute to the maintenance of maternal sleep difficulties symptoms (measured at T1 and T2). The second aim was to explore the associations among PTS symptoms, maternal sleep difficulties, and the three dimensions of parenting stress (PD, PCDI, and DC) at 3 months postpartum (T2) by examining the mediational role of both sleep difficulties and childbirth-related PTS symptoms.

Regarding the first aim, the mediational results confirmed the hypothesis (H1) that sleep difficulties during pregnancy (T0) and 1 month after childbirth (T1) could mediate the associations between PTS symptoms (at T0 and at T1) as well as the associations between PTS symptoms (at T1 and at T2). In the same way, results indicated PTS symptoms during pregnancy (T0) and 1 month after childbirth (T1) mediated both the associations between maternal sleep difficulties (at T0 and at T1) and the associations between maternal sleep symptoms (at T1 and at T2). In other words, it is possible to confirm the bidirectional effects between maternal sleep difficulties and PTS symptoms and their reciprocal role of maintenance of symptoms. Specifically, results showed that the total variance accounted for in the models performed was larger for the sleep difficulties as maintaining factors for ongoing PTS symptoms.

These results are consistent with the literature (Koren et al., 2002; Lamarche and De Koninck, 2007; Mellman et al., 2007; Garthus-Niegel et al., 2015), showing that sleep difficulties could be associated with subsequent PTS disorder. The linking between sleep disorders and PTSD was based on the assumption that impaired sleep (insufficient or fragmented sleep) could hinder an individual’s ability to manage stress and recover from traumatic events (Williams et al., 2015). More specifically, a series of studies indicated that poor quality of sleep could reduce the cognitive resources available to manage stress, create a state of hyperarousal, and hinder restorative sleep for rebalancing the mental state after the traumatic experiences (Mellman et al., 2007; Bryant et al., 2010; Garthus-Niegel et al., 2015; Williams et al., 2015). Moreover, Garthus-Niegel et al. (2015) outlined that the burden of insomnia may exacerbate postpartum PTSD symptoms and diminish the women’s skills to manage these symptoms, while Mellman et al. (2007) outlined that REM sleep and higher dream activity in the initial REM period could facilitate adaptive processing of distressing emotions, which may be critical to recovery from traumatic events. Conversely, fragmented REM sleep, expressed by sleep-disordered breathing, nightmares, or insomnia, could reduce this rebalancing ability and facilitate the development and persistence of PTS symptoms.

Regarding the second aim, the mediational analyses of the participants at 3 months postpartum (T2) confirmed our second hypothesis (H2). More precisely, in line with the literature (McDonald et al., 2011; Camisasca et al., 2017; Di Blasio et al., 2017), PTS symptoms were significantly associated with the three dimensions of parenting stress. Also, a significant association between maternal sleep difficulties and parenting stress was found, consistent with the literature (Kahn et al., 2013; Tikotzky, 2016), which outlined how maternal sleep difficulties could lead to more negative maternal emotions and perceptions in mothers’ relations with infants, strictly associated with the construct of parenting stress.

Regarding our Hypothesis 3 (H3), the results indicated the presence of bidirectional effects between PTS symptoms and maternal sleep difficulties in their associations with parenting stress. In particular, the results indicated that sleep difficulties mediated the links between PTS symptoms and all three dimensions of parenting stress, while PTS symptoms partially mediated the links between sleep difficulties and the PD and difficult-child (DC) dimensions.

To date, no empirical studies have explored the associations between maternal sleep difficulties and parenting stress. Therefore, we could explain our data by focusing on the literature concerning the associations among sleep disturbances and emotional functioning (Kahn et al., 2013). More specifically, according to this literature, we could suppose that women with sleep difficulties may have increased tension and anxiety, confusion, fatigue, and mood disturbances (Paterson et al., 2011), significantly associated with feelings of incompetence, insecurity, and depression, as included in the PD dimension. It was also suggested (Kahn et al., 2013; Tikotzky, 2016) that poor maternal sleep leads to deficits in emotion regulation, with consequent negative perceptions in terms of the emotional quality of their relationship with their children and of their children’s behaviors, which are significantly associated with the PCDI and DC dimensions.

The mediational role of childbirth PTS symptoms in the associations between sleep difficulties and parenting stress is also consistent with previous literature (McDonald et al., 2011; Camisasca et al., 2017; Di Blasio et al., 2017) outlining how childcare could be a rather difficult challenge for women with more PTS symptoms, resulting in parenting stress. The results also indicated that PTS symptoms did not mediate the direct effects of maternal sleep difficulties on the PCDI dimension of parenting stress. This finding needs further studies to be substantiated, to better explore the associations between PTS symptoms and this dimension of parenting stress.

However, we could suppose that mechanisms besides PTS symptoms could intervene in the insurgence of the PCDI dimension. For example, we could hypothesize that more problematic maternal bonding resulting from sleep disturbances (Tikotzky, 2016) could specifically affect the PCDI dimension, which refers to the mothers’ perceptions and feelings about their relationship with their children. More precisely, we could suppose that the maternal perceptions and feelings of satisfaction, happiness, and gratification, which specifically result from the quality of interactions with children (PCDI), could be strictly affected by the quality of the maternal bonding (the mother’s feelings toward her infant and perceptions of her emotional relationship with the infant).

There are some limitations to this study. The first limitation is to have collected only self-reported data, which may be subject to social desirability and cannot be verified independently. Objective measures of sleep (e.g., actigraphy; Tikotzky and Sadeh, 2001; Dørheim et al., 2009) and observational measures of the mother–infant relationships are necessary to replicate and better understand our results. The small number of participants may limit the generalizability of our results, even though this limitation was partly overcome by the use of the bootstrapping procedure for the mediational analyses. Moreover, the relatively low prevalence of women scoring in the clinical ranges for PTSD and insomnia prevents the possibility that our findings could be generalized to clinical populations. Furthermore, we did not explore the possible role of other potential maintaining factors such as social support, marital quality, personality, or maladaptive coping. Another limitation concerns the LASC measure, which has not been validated in Italian, even though the scale has demonstrated good reliability and validity in past research (Di Blasio et al., 2015b). Moreover, PTSD in pregnancy was assessed in relation to any traumatic event, while after birth, the PTSD was examined only in relation to the birth. According to Ayers and Pickering (2001, p. 116), “the lower specificity of the measure used in pregnancy suggests that other psychiatric disorders or distress may have inflated the rate of PTSD found in pregnancy.”

## Conclusion

Our study contributes to the understanding of the maintenance factor of PTS symptoms by indicating that the quality of sleep among mothers during the postpartum period could be associated with PTS symptoms and the perceptions and feelings of their interactions with their infant. As maternal sleep disturbances have received little attention in relation to mother–infant relationships, the present study highlights the importance of exploring the negative effects of maternal sleep disorders. Future research directions involving clinical samples could better investigate the predictive effects of PTS symptoms on sleep difficulties and of maternal difficulties on PTS symptoms, particularly regarding their associations with parenting stress, to better substantiate our results.

## Author Contributions

PDB developed the study concept and created the study design. EC performed the data analysis and interpretation under the supervision of PDB. EC and SM drafted the manuscript. PDB provided critical revisions. All authors approved the final version of the manuscript for submission.

## Conflict of Interest Statement

The authors declare that the research was conducted in the absence of any commercial or financial relationships that could be construed as a potential conflict of interest.
